# Design and Pilot Evaluation of an IoT-Based Blood Pressure Monitoring System for Rabbits

**DOI:** 10.3390/bioengineering13040384

**Published:** 2026-03-26

**Authors:** Carlos Exequiel Garay, Gonzalo Nicolás Mansilla, Rossana Elena Madrid, Agustina González Colombres, Susana Josefina Jerez

**Affiliations:** 1 CIASUR (Centro de Investigación de Atmósfera Superior y Radiopropagación), Facultad Regional Tucumán (FRT), Universidad Tecnológica Nacional (UTN), Rivadavia 1050, San Miguel de Tucumán 4000, Argentina; exequielgaray@doc.frt.utn.edu.ar (C.E.G.); mansillagn@gmail.com (G.N.M.); 2 Laboratorio de Medios e Interfases (LAMEIN), Departamento de Bioingeniería (DBI), Facultad de Ciencias Exactas y Tecnología (FACET), Universidad Nacional de Tucumán, San Miguel de Tucumán 4000, Argentina; rmadrid@herrera.unt.edu.ar; 3 Instituto Superior de Investigaciones Biológicas (INSIBIO), Consejo Nacional de Investigaciones Científicas y Técnicas (CONICET), San Miguel de Tucumán 4000, Argentina; agustinagcol@hotmail.com

**Keywords:** Internet of Things (IoT), blood pressure monitoring, preclinical models, edge computing, cloud computing

## Abstract

Telemedicine, driven by the Internet of Things (IoT) and wireless connectivity, is essential for managing cardiovascular diseases, where hypertension remains the primary risk factor. In preclinical research, rabbits are superior biological models compared to rodents due to their human-like lipid metabolism. However, continuous blood pressure monitoring in this species remains challenging. The gold-standard technique (direct carotid catheterization) requires terminal procedures, and indirect methods (Doppler, oscillometric) show limited agreement with direct measurements. Furthermore, commercially available implantable telemetry platforms, while enabling real-time monitoring in freely moving animals, require costly surgical implantation, specialized proprietary hardware, and post-operative recovery periods that may confound early hemodynamic data. To address these limitations, this study presents a low-cost, customizable, and minimally invasive monitoring system utilizing a pressure transducer in the central auricular artery. The device integrates an ESP32 microcontroller with IoT technology for digital signal processing and seamless wireless data transmission to the ThingSpeak cloud platform. Unlike implantable telemetry, the proposed approach avoids surgical implantation and its associated costs and recovery time, while still enabling continuous, real-time hemodynamic tracking throughout the experimental period. A pilot evaluation against the BIOPAC MP100 reference (carotid artery) demonstrated relative errors of 1.60% for mean arterial pressure, 8.58% for systolic blood pressure, and 2.43% for diastolic blood pressure. By reducing invasiveness and enhancing remote data accessibility, this system provides a promising framework for the preclinical evaluation of antihypertensive agents and cardiovascular mechanisms, bridging the gap between edge computing and remote clinical diagnostics.

## 1. Introduction

In rabbit production systems (commercial farms and laboratory animal facilities), continuous supervision is often limited, whereas rapid microenvironmental fluctuations (e.g., temperature, humidity, ventilation) and early changes associated with health status can progress into stress, disease, and losses if not detected promptly. In high-density settings, manual inspection is intermittent and does not ensure early detection. Therefore, an IoT-based approach—utilizing distributed sensors and/or non-intrusive perception, low-bandwidth connectivity for periodic updates, and cloud services for logging and alerts—is key to enabling continuous, scalable monitoring and improving veterinary and husbandry decision-making under operational constraints related to maintenance and connectivity availability [1]. For these reasons, it is essential to explore efficient wireless connectivity alternatives.

The Internet of Things (IoT) is one such alternative. It refers to the digital interconnection of objects, people, and even animals to the internet. The concept was introduced by Kevin Ashton in 1999 with the invention of Radio Frequency Identification (RFID) [2]. As of recent estimates, over 20 billion objects are connected to the internet, a figure projected to reach 40.6 billion by 2034.

Therefore, it is important to consider the limitations of these devices in terms of storage, processing, computing, and security capabilities [3].

When IoT technologies are applied to healthcare, the cost and efficiency of medical services can be improved and expanded by automating tasks that were previously performed by humans [4]. To fulfill this objective, it is necessary to implement wireless technologies that are efficient in both energy consumption and data transmission. These technologies must meet various requirements, including reliability, interoperability, energy efficiency, low-latency responses, mobility, and security.

Most animal health monitoring systems are implemented as wearable devices that use short- or medium-range communication technologies to transmit data to the cloud. Several studies have addressed animal monitoring from different technological perspectives. Recent work in smart agricultural monitoring has shown that the selection of the wireless link should be driven by application requirements such as coverage, energy consumption, and data rate; in this context, Wi-Fi, Zigbee, and LoRaWAN remain practical alternatives for IoT deployments [5]. In parallel, wearable IoT approaches have expanded the possibilities for continuous and remote animal surveillance in precision livestock systems [6].

Within healthcare-oriented IoT systems, the communication and computing architecture must also support timely transmission, interoperability, and remote monitoring. Recent reviews highlight that these design choices are central to reliable physiological monitoring platforms that integrate sensors, embedded devices, and cloud services [7].

Wearable devices for animal health monitoring enable the measurement of physiological parameters such as blood pressure, heart rate, respiratory rate, and other vital signs, thereby assisting caregivers in the early detection and prevention of serious diseases [8]. For instance, Bello and Passaglia [9] developed a wireless sensor for continuous intraocular pressure monitoring in conscious mice, transmitting data via Bluetooth. Similarly, Chen et al. [10] proposed a telemetry system for measuring body temperature in rabbits, which transmits the measurements to a computer for storage and analysis.

Monitoring and controlling blood pressure in animals is essential for the early detection of cardiovascular diseases and for monitoring medical and experimental treatment. In rabbits, blood pressure is a key indicator of health and well-being, as they are prone to cardiovascular conditions [11]. The reference technique (gold standard) for measuring blood pressure in rabbits is direct measurement using a pressure transducer, which involves carotid artery catheterization followed by euthanasia. The most commonly used indirect methods today include Doppler, oscillometric, and plethysmographic techniques [12,13,14,15]. Commercial implantable telemetry platforms for laboratory animals, such as DSI PhysioTel [16] and emka easyTEL+ [17], already enable continuous arterial pressure recording in conscious, freely moving animals. However, these systems require surgical implantation of the transmitter, which entails significant costs, specialized surgical infrastructure, post-operative recovery periods that may confound early hemodynamic data, and the risk of surgical complications. Furthermore, their proprietary hardware and software architectures limit customization and integration with open IoT platforms. Among the indirect (non-invasive) techniques, only the Doppler method has demonstrated some correlation with the direct method (Gold standard). Therefore, the contribution of the present study is not the general concept of continuous monitoring itself, but the development of a low-cost, customizable IoT-based alternative for rabbits that uses minimally invasive catheterization of the central auricular artery and integrates ESP32-based acquisition with ThingSpeak cloud services for remote visualization and analysis. Unlike implantable telemetry, this approach avoids surgical implantation and its associated costs and recovery time, while unlike indirect methods, it provides direct arterial pressure measurements with beat-to-beat resolution. Given the aforementioned considerations, it is crucial to investigate the implementation and performance of IoT in the field of animal health for the early detection of chronic diseases.

This study contributes by presenting an IoT model designed for monitoring blood pressure in rabbits, structured across four distinct layers: the cloud layer, the edge layer, the physical layer, and the network layer [18]. This technological development enables the monitoring of pressure changes in an experimental animal (specifically a rabbit) over the course of the experimental period and facilitates the evaluation of various therapeutic interventions, allowing for preclinical assessment of antihypertensive agents.

## 2. Materials and Methods

**Figure 1:**
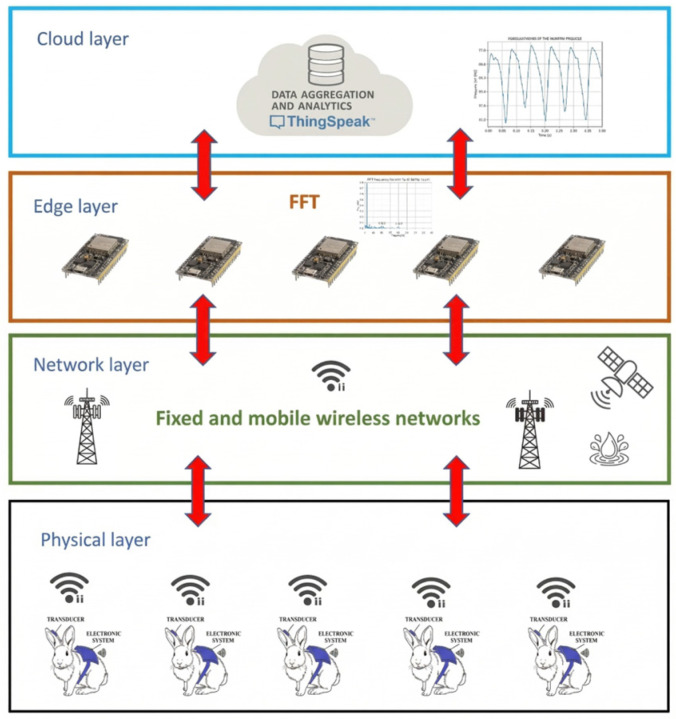
Layers of the system.

In the developed four-layer system, the cloud layer is represented by ThingSpeak (MathWorks, 2025), an open-source platform for the Internet of Things (IoT) that enables the collection, storage, analysis, and visualization of data from sensors connected to the internet [19]. The edge layer is where user-level computational activities are enabled. Specifically, it is where the Fast Fourier Transform (FFT) of the rabbit’s physiological parameters is performed (see Figure 1).

The physical layer is responsible for activating the rabbits’ blood pressure sensor (PX260 TruWave Disposable Pressure Transducer, Edwards Lifesciences, Irvine, CA, USA) to collect data. These data are then made available for processing and for generating information about the animals’ health status. The network layer provides the necessary infrastructure to enable data communication and overall connectivity. In the present implementation, this layer relies on Wi-Fi connectivity available in the animal facility to transmit the digitized pressure signal from the ESP32 to the ThingSpeak platform. This choice is consistent with IoT monitoring architectures that combine practical wireless links with cloud-based data visualization and storage [5,7].

### 2.1. System Architecture

Billions of internet-connected devices, such as wearables with limited hardware capabilities, possess insufficient local storage for the data they generate. One approach to address this limitation is to transfer the data to the cloud for storage and processing. However, due to the massive number of devices and their limited processing power and bandwidth, cloud-based latency can be significantly high. To mitigate this issue, fog or edge computing provides the necessary capabilities to offer an effective solution.

Fog Computing is a “system-level architecture that distributes resources and services of computing, storage, control, and networking anywhere along the continuum from the Cloud to Things” [20]. Fog computing is essential for emerging IoT applications (such as industrial automation, transportation, medical devices, etc.) that demand real-time or predictable latency. Due to its geographic distribution, fog computing is well-suited for locally processing information generated by IoT nodes, enabling applications that require low latency [21].

Cloud services include data processing, storage, and analysis, as well as security, privacy, alert and notification services, device management, content management, user services, and more [22]. In the present case, the cloud service is implemented using ThingSpeak [19].

### 2.2. System Development

**Figure 2:**
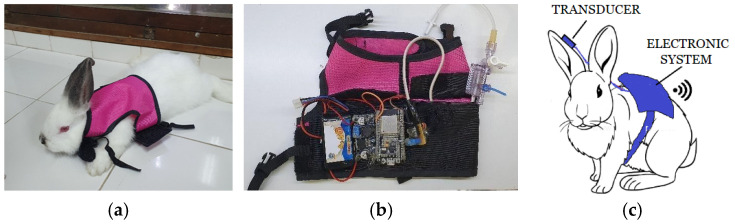
(**a**) Backpack installed on one of the rabbits housing the measurement system; (**b**) Electronics of the measuring system; (**c**) The system placed on the rabbit.

The blood pressure monitoring system consisted of a vest adapted to the rabbit’s body (Figure 2a), with a pocket to house the pressure transducer and another pocket containing the electronic system (Figure 2b). The blood pressure sensor, in this case, the PX260 TruWave Disposable Pressure Transducer (Edwards Lifesciences, Irvine, CA, USA) consists of a matrix of strain gauges arranged in a Wheatstone bridge configuration. The output voltage varies according to the pressure applied to the transducer, resulting in a voltage change at the two output terminals of the Wheatstone bridge. Figure 2c shows a schematic of the developed system positioned on the rabbit.

The sensor output is amplified using the INA122 instrumentation amplifier (Texas Instruments, Dallas, TX, USA), a precision instrumentation amplifier, to elevate the voltage level to a range detectable by the analog-to-digital conversion (ADC) module of the ESP32 microcontroller (Espressif Systems, Shanghai, China). The amplification circuit follows the configuration recommended in the INA122 datasheet [23] for amplifying signals from a Wheatstone bridge, with the addition of a capacitor connected between the output of the IC and ground. The inclusion of this capacitor, recommended by the ESP32 manufacturer, serves to reduce noise that may be picked up by the ADC module [24].

**Figure 3:**
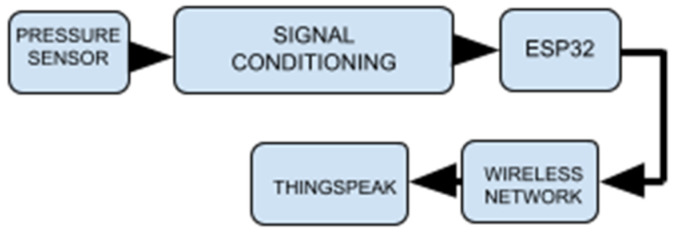
Block diagram of the system.

Figure 3 presents a block diagram of the implemented system.

**Figure 4:**
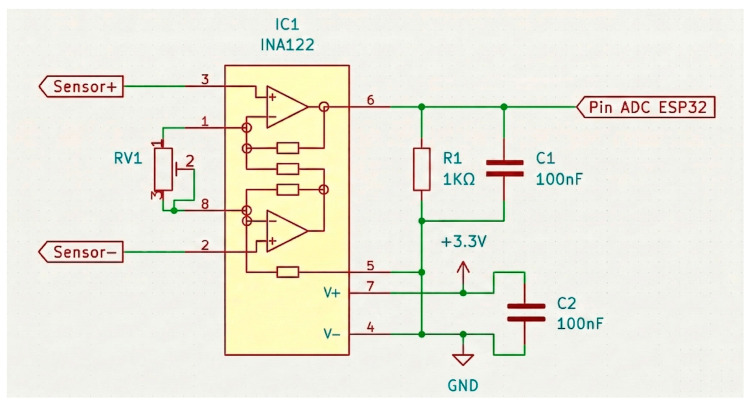
Instrumentation amplifier circuit.

The circuit of the implemented instrumentation amplifier is shown in the following Figure 4:

The ESP32 microcontroller captures the signal from the amplifier at a sampling frequency of 1000 Hz, acquiring approximately 10,000 samples to obtain several cycles of the rabbit’s heartbeat (3–6 Hz). Finally, the samples are transmitted to the ThingSpeak platform via a wireless connection, where the necessary procedures are performed to present the acquired data in a simple format for the relevant personnel. From this dataset, the systolic and diastolic pressure values are extracted, and the following formula is applied to determine the mean arterial pressure (MAP):

(1) $$MAP=\frac{\left(2\cdot DBP\right)+SBP}{3}$$

where DBP is the diastolic blood pressure and SBP is the systolic blood pressure.

Once these results are obtained, they are transmitted via the internet to the ThingSpeak platform for subsequent analysis by the end user. The ESP32 microcontroller is programmed to perform this procedure at various time intervals, starting from 1 min and extending as needed. In this case, the microcontroller is configured to execute the measurement routine either every 12 h or every 10 min, depending on the specific requirements for monitoring the rabbit’s behavior.

To calculate and measure time intervals, the microcontroller relies on its internal clock. However, due to fluctuations in its operating frequency, temporal estimation errors are introduced. These errors accumulate over time, potentially causing significant deviations between the user-defined wait time and the time estimated by the device. To compensate for this drift, the system periodically (every 1 h) queries an NTP (Network Time Protocol) time server as a correction and synchronization mechanism. A time server is a computer with access to a reference clock, used to synchronize the date and time of devices connected to a network [25], while NTP is the network protocol used to transmit this information [26]. This ensures that measurements are performed at the pre-established times.

Moreover, since the ESP32 remains idle for most of the time while waiting for these intervals to elapse, the device’s SLEEP modes were utilized to increase energy efficiency by reducing current consumption. SLEEP modes are strategies used to minimize the energy consumption of a microcontroller when it is not actively performing tasks. In this work, the DEEP-SLEEP mode of the ESP32 is utilized, which powers down the processor, RAM, and all internal modules of the device, except for those necessary to enable system wake-up.

Exit from this state occurs through an external event or an interrupt generated by a module that remains active and functions as the system’s “wake-up” trigger. Specifically, the RTC (Real Time Clock) module is employed. In addition to its primary role of maintaining time and date, the RTC includes a configurable internal timer. This timer allows the setting of a wait interval after which a countdown begins; once it reaches zero, an event is generated that wakes the ESP32 from DEEP-SLEEP mode and restarts program execution from the beginning.

Additionally, the RTC includes a small backup RAM that remains powered during DEEP-SLEEP mode. This feature is particularly useful, as it allows the storage of relevant information prior to entering this mode, preventing the loss of variables due to the power-down of the main RAM. Consequently, critical data required for proper program execution upon wake-up are preserved, circumventing the inherent memory limitations of the DEEP-SLEEP mode [27].

**Figure 5:**
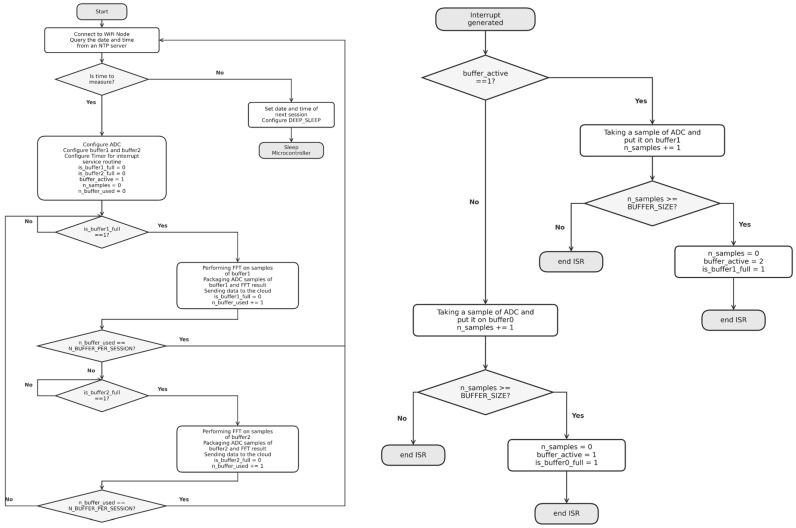
Flowchart of firmware implementation.

Finally, the acquired samples are packaged and transmitted to a cloud service for subsequent storage and processing. The flowchart of the firmware implementation is shown in Figure 5.

### 2.3. Rabbit Preparation

For this study, four male hybrid rabbits (a cross between Californian and New Zealand breeds), weighing between 2 kg and 3 kg, were used. The animals were obtained from the rabbit production unit of the Faculty of Agronomy and Animal Science at the National University of Tucumán (UNT) and housed in the rabbit vivarium of INSIBIO. A sample size of N = 4 rabbits was selected based on the 3Rs principle (Replacement, Reduction, and Refinement). This approach aims to minimize the number of animals subjected to experimentation in compliance with international guidelines, such as those established by the American Association for Laboratory Animal Science (AALAS). Given that the objective is to demonstrate technical feasibility—specifically, the functionality of the sensor and the physical viability of the procedure—the use of a minimal sample size is ethically superior to employing a larger cohort prior to confirming initial efficacy. The vivarium is a facility that houses animals with defined genetic and microbiological quality, used for research purposes. All animal care and use programs were performed according to the Guide for the Care and Use of Laboratory Animals (NIH Publication 8th edition, updated 2011). The protocols were unanimously approved by the Institutional Committee for the Care and Use of Laboratory Animals (CICUAL) of UNT under research protocol No. 076/2023, dated 2 June 2023.

Prior to housing the rabbits in the quarantine room, a thorough cleaning and disinfection of cages, floors, and surfaces were carried out. Upon arrival at the vivarium, the rabbits were placed individually in metal cages, equipped and integrated into a micro-ventilated rack system that complies with international standards on animal welfare and biosafety. The animals remained under quarantine for 15 days to rule out any pathologies, stabilize immune function, and perform internal parasite examinations.

During this period, detailed records were maintained regarding the date of entry, previous illnesses, treatments, body weight, and behavioral assessments. The animals were housed under a 12 h light/12 h dark cycle, controlled by a mechanical timer. Ambient temperature was maintained at 24 ± 1 °C using air conditioning and monitored with a wall-mounted thermometer.

Daily maintenance included changing bedding, removing soiled wood shavings with feces and urine, disinfecting trays, and refilling with clean shavings. Each day, the rabbits were weighed, their general condition assessed, and their water and food intake measured [28]. Environmental enrichment was implemented through weekly dietary supplementation with fresh fruit (apples or carrots) and a once-weekly free-roaming period to promote natural exploratory behavior.

### 2.4. Experimental Procedures

The experiments were conducted over a one-month period. Initially, the performance of the proposed system was assessed. Subsequently, the system was validated by comparing the obtained data with those from the reference system: direct blood pressure measurement.

**Figure 6:**
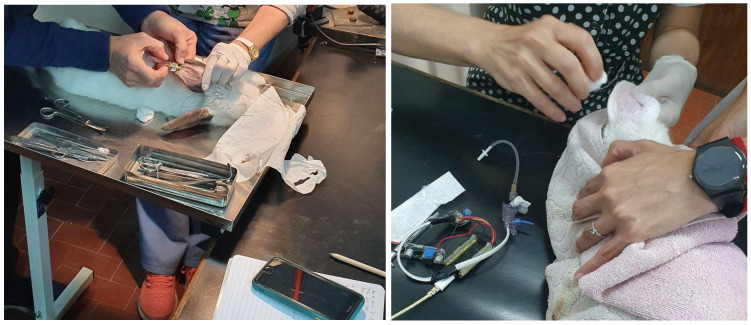
Procedure for intraarterial catheter placement for the proposed system.

The experimental subjects were divided into two groups, each consisting of two rabbits. In the first group, the performance of the proposed system was assessed by placing the pressure transducer in the central auricular artery of the rabbit’s ear (Figure 6). The catheterization was optimized using an Abbocath-T 24 G catheter (ICU Medical, San Clemente, CA, USA), which remained in place throughout the experimental period. Rabbits were immobilized using a restraint device specifically designed at INSIBIO-CONICET for performing experimental procedures. During the 15 days prior to the procedure, the animals were placed in this device daily at the same time to acclimate them and reduce stress, which could otherwise alter the blood pressure values.

The procedure was as follows: after the auricular artery was visually identified, the inner surface of the ear was shaved, disinfected with 5% povidone-iodine (Pervinox povidone-iodine antiseptic solution (Laboratorio Elea Phoenix S.A., Buenos Aires, Argentina), and locally anesthetized using 7% lidocaine gel. The catheter was then inserted and secured with hypoallergenic adhesive tape. The animal’s behavior in response to the foreign object was monitored every four hours during the first 48 h. If the catheter remained viable after this period, the blood pressure measurement was conducted.

To perform the measurement, the rabbit was placed in the immobilization device, the catheter was connected to the pressure transducer, and the arterial pressure signal was captured and transmitted to the cloud. Each acquisition consisted of 10,000 samples at a sampling rate of 1 kHz, yielding a 10 s recording window that captured several complete cardiac cycles (resting heart rate in rabbits: approximately 3–6 Hz). This procedure was carried out on two rabbits from the first group.

In the second group, composed of two additional rabbits, the data obtained from the first group were validated by comparing them with the gold standard blood pressure measurement technique, applied to the carotid artery.

The procedure was as follows: Animals were fasted for 8 h, weighed, and their vital signs were monitored. Pre-anesthesia was administered subcutaneously using diazepam at a dose of 5 mg/kg. Anesthesia was then induced via a subcutaneous injection of ketamine (50 mg/kg) [29]. The neck was shaved in the region of the carotid artery. Subsequently, 7% lidocaine was injected subcutaneously prior to the first skin incision. Using a scalpel, an incision was made at the level of the carotid artery, which runs parallel to the trachea. The skin, muscle, and fat layers were dissected until the artery was located. Surrounding fat and nerves were cleared, and a small incision was made in the artery to insert the catheter. The catheter was secured with sutures, and a temporary clamp was applied to momentarily obstruct blood flow.

Simultaneously, the same procedure as that used for the first group was applied: catheterization of the auricular artery was performed, and the pressure transducer (Edwards Px260) was connected to transmit data to the cloud.

Finally, the carotid artery catheter was connected to a pressure transducer (Gould, Eichstetten, Germany), the arterial clamp was removed, and blood pressure readings were acquired using the BIOPAC MP100 data acquisition and analysis system.

**Figure 7:**
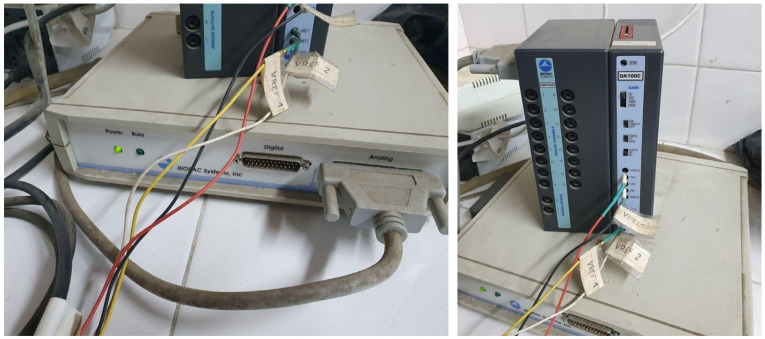
Reference device BIOPAC model MP100.

This system was specifically designed for measuring biological signals and features a 12-bit resolution D/A converter [30]. The system is connected to a PC, as shown in Figure 7. Thus, simultaneous blood pressure values were obtained from both the auricular and carotid arteries for comparative analysis. Animals subjected to direct arterial blood pressure measurement via carotid artery cannulation were euthanized by exsanguination while under deep anesthesia. This endpoint is commonly utilized in studies involving such hemodynamic determinations. The other two animals were housed in the vivarium for use in future experimental procedures.

During each validation session, five non-overlapping acquisition windows of 10,000 samples each (10 s at 1 kHz) were collected simultaneously from the auricular artery (proposed IoT system) and the carotid artery reference (BIOPAC MP100 data acquisition system, BIOPAC Systems, Inc., Goleta, CA, USA) while the animal remained under stable anesthesia. Since the carotid artery cannulation required for gold-standard comparison is a terminal procedure, only one session per rabbit was possible. With two rabbits in the validation group, this protocol yielded a total of N = 10 paired recordings (5 windows × 2 rabbits), which formed the basis of the agreement analysis reported in Section 4.

## 3. Results

### 3.1. System Calibration

**Figure 8:**
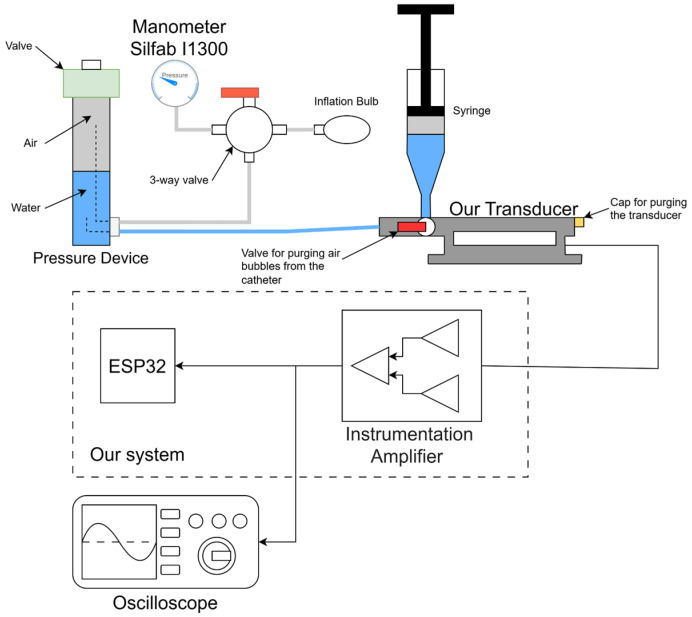
Hydraulic system used for calibrating the system.

To calibrate the system, the pressure transducer is connected to an external hydraulic system, shown in Figure 8, which generates step pressure. Initially, water was introduced into the system (Figure 8). Subsequently, any air bubbles introduced during this step were expelled using a syringe and the transducer purge cap. The desired pressure is generated within the pressure device by introducing air into it using an inflation bulb and a manometer Silfab I1300 aneroid sphygmomanometer (Silvestrin Fabris S.R.L., Buenos Aires, Argentina). Subsequently, the valve is actuated to produce a step pressure that is applied to the transducer. Finally, the output signal from the instrumentation amplifier is acquired in order to perform system calibration. The transducer is calibrated by measuring its response to steps pressure of different amplitudes, from 15 mmHg to 85 mmHg with a step of 5 mmHg. By applying the filtering technique that will be described later in Section 3.2, the ADC count corresponding to the peak of the response—associated with the amplitude of each step pressure—is obtained. To determine the calibration coefficients, linear regression is applied to fifteen data points relating pulse amplitude to ADC counts, thereby estimating the offset and scale required to convert ADC counts into pressure. An oscilloscope was connected to the output of the instrumentation amplifier to visualize the output signal and verify proper system operation.

The calibration coefficients are stored in a cloud-side table along with the date and time at which they were obtained, as well as their corresponding expiration date and time, defined by the completion of the pressure measurement of the rabbit for that session.

This procedure was performed prior to each measurement session of each rabbit.

**Figure 9:**
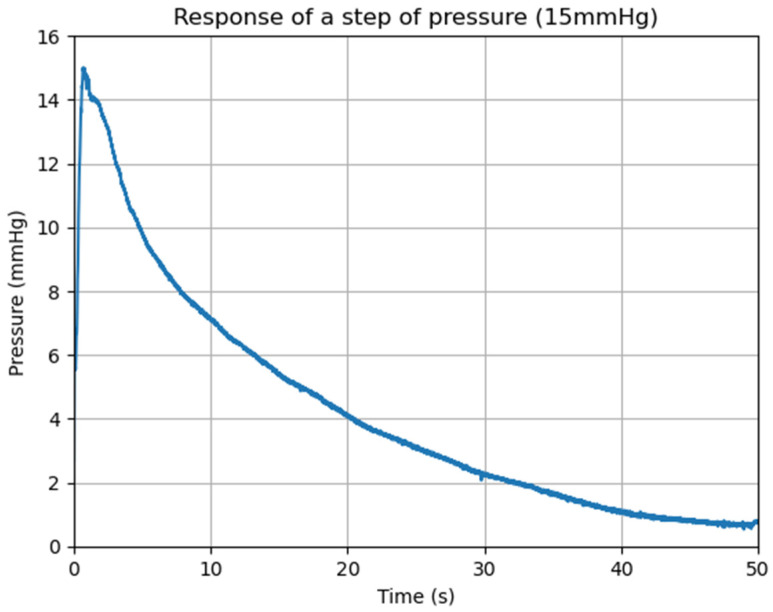
System response to a step pressure of 15 mmHg.

Figure 9 represents the transducer response of a step pressure with an amplitude of 15 mmHg.

### 3.2. Data Acquisition and Processing

To maintain independence between the microcontroller’s firmware and the sensor/amplification stages, the system transmits the raw ADC count directly to the cloud.

Conversion and calibration coefficients are then applied in the cloud, resulting in a modular firmware design that is not tied to a specific sensor and/or amplifier.

**Figure 10:**
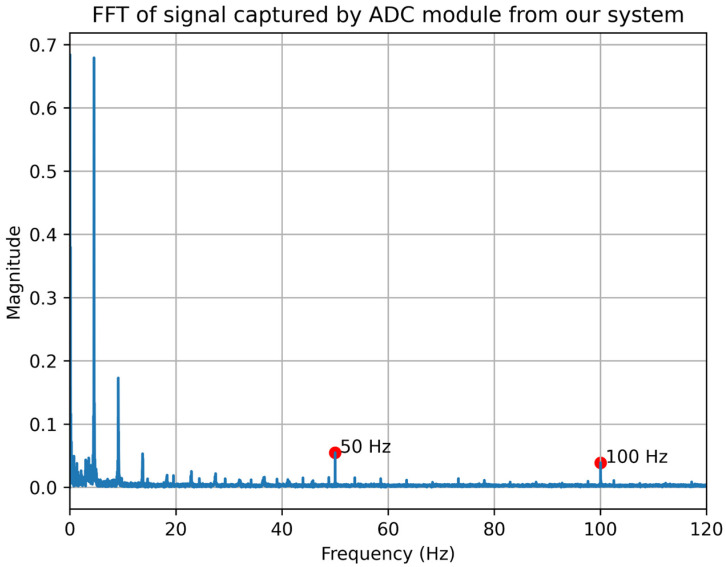
FFT of the rabbit’s blood pressure signal captured by ADC module of the developed system.

Interference from the electrical power grid constitutes a common source of external noise in devices operating with millivolt-range transducers [31,32]. Figure 10 shows the FFT of the rabbit’s blood pressure signal captured by the ADC module, where peaks can be observed at the power line frequency and its first harmonic—50 Hz and 100 Hz in this case.

Since the frequency range of interest is lower than that of the power grid, a filter can be applied to reduce the noise caused by this interference. A digital filter offers the advantage of being immune to noise, as it is implemented via software or digital circuits [33]. Therefore, it is not affected by electrical noise from the power grid.

The implemented filter is a second-order IIR Butterworth filter, selected for its maximally flat response in the passband [34], with a cutoff frequency of 20 Hz. To eliminate signal distortion caused by filtering transients, a forward–backward filtering technique was applied [35]. This filter is applied on the cloud side to the set of samples that were packaged and transmitted to the server.

The ThingSpeak platform sends, receives, and stores data in character format. For example, to send the value 10 to ThingSpeak, it must first be converted into a character string, which is then transmitted over the internet using an application protocol such as HTTP or MQTT [19]. ThingSpeak organizes incoming data using channels, each of which can store up to 8 fields of data. Following this structure, storing 10,000 samples would require 1250 channels. However, the academic and standard licenses of ThingSpeak allow the creation of only up to 250 channels, meaning that five licenses would be needed. Additionally, the firmware would have to be programmed with 1250 channel identifiers along with their respective write keys in order to transmit the data.

To reduce the number of channels required without decreasing the volume of data sent, a data-packing strategy was devised. This method allows multiple data points to be encoded into a single field, thereby drastically reducing the number of channels required. ThingSpeak stores the numerical data in each field as character strings, with a maximum length of 255 characters. This feature allows multiple samples to be grouped within a single field, which can later be unpacked in the cloud to retrieve the original dataset. Since the maximum value produced by the ADC module is 4095, each sample is represented as a 4-character string. These strings are concatenated sequentially up to a total length of 252 characters—without exceeding the platform’s field limit—allowing 63 samples per field and a total of 504 samples per channel.

**Figure 11:**
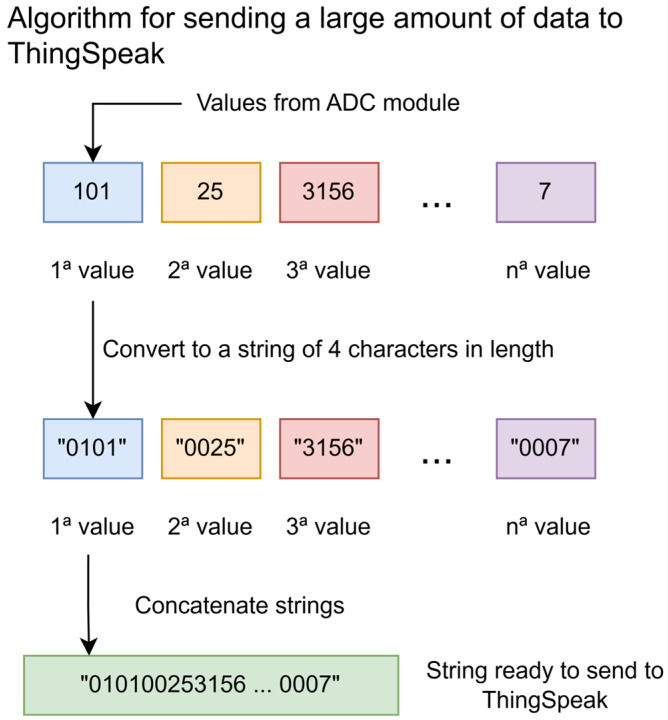
Algorithm for sending a large amount of data to Thingspeak.

Using this method, 10,080 values can be stored using only 20 channels, significantly reducing the number of channels required while preserving the existing workflow and functionality of ThingSpeak. Subsequently, to reconstruct the original dataset, the reverse process is applied. First, the character string stored in each field is retrieved and split into 4-character substrings. Each substring is then converted back into its numerical format, and the resulting values are sequentially grouped to recover the originally acquired numerical dataset (Figure 11).

The “format” of the data sent to ThingSpeak is not natively recognized by the platform; therefore, it is necessary to implement a script or program to interpret the data and display it graphically. Within each channel, ThingSpeak provides a space to execute MATLAB code (MATLAB R2025b, MathWorks, Natick, MA, USA), which can be used to create “interactive windows” such as line plots, bar charts, histograms, etc., using the data sent to the platform. Through this functionality, it is possible to develop a script that retrieves the character strings from the used channels to reconstruct the original sequence of values. Consequently, the recovered data can be filtered, graphically represented, or processed to extract parameters of interest, such as systolic and diastolic pressure, from the signal captured by the device.

To improve the overall system performance, part of the processing was moved to the edge (ESP32 microcontroller), while another part remains in the cloud (ThingSpeak). The edge, which is responsible for managing the pressure sensor operation, is also where the Fast Fourier Transform (FFT) is performed on the data. This allows only the necessary information for visualizing the frequency spectrum of the captured signal to be transmitted to the cloud. Since it is not essential to capture all signal details for frequency spectrum analysis—and considering that the device measures biological signals, which are typically low-frequency—a reduction in the sampling rate to 250 samples per second was applied. This ensures capture of the signal’s frequency spectrum, as well as the 50 Hz and 100 Hz noise components from the power grid (50 Hz in this case and its first harmonic).

**Figure 12:**
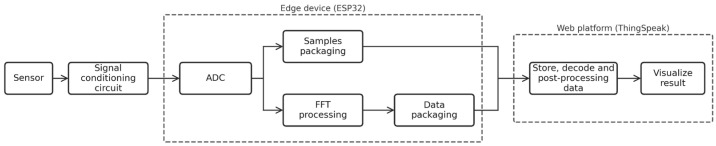
Signal processing pipeline implemented by our system.

**Figure 13:**
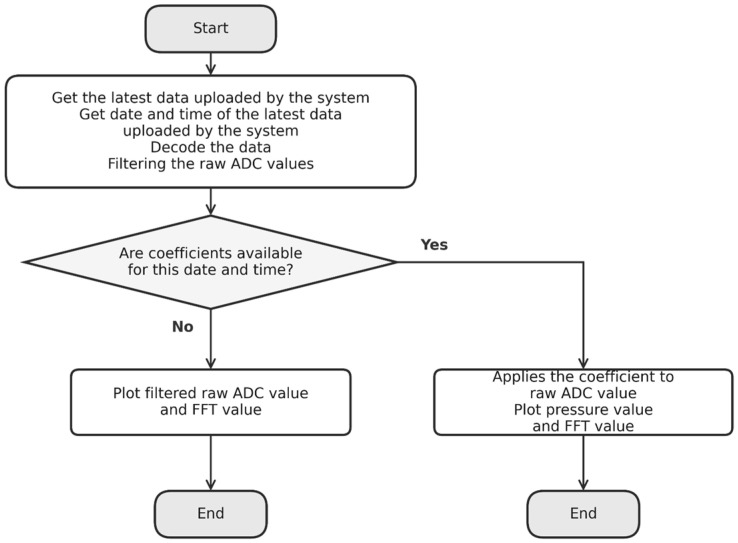
Flowchart of MATLAB code on ThingSpeak.

Furthermore, because the FFT of the captured signal is consistent regardless of the system’s application, this processing can be performed on the device without compromising firmware modularity. The FFT results are transmitted to ThingSpeak in the same manner as the raw signal values, with the difference that each data string begins with a 1 to indicate a negative value or a 0 for a positive value. The amplitude values are limited between −100 and 100, and only one decimal digit is included. This results in 1 character for the sign indicator, 3 characters for the integer part, and 1 character for the decimal part—so each FFT value occupies 5 characters. Figure 12 shows the signal processing pipeline implemented by the system and the web platform. Figure 13 shows the flowchart of MATLAB code on ThingSpeak.

The data are later adjusted in the cloud according to the system’s calibration coefficients, and the corresponding graph is generated. Although there are algorithms available for data compression—both lossy and lossless—that reduce the number of bytes required and allow for more data to be packed into a single transmission [36,37,38], their implementation typically requires prior knowledge of the data to be compressed. In the case of lossy algorithms, it is necessary to determine whether the algorithm can reconstruct the original signal in a way that allows for equivalent analysis as when using a reference instrument. For lossless algorithms, it is important to assess whether the nature of the data justifies the additional processing required, both at the cloud and edge levels, and whether the reduction in data size warrants the extra computational effort.

Although reference instrument data were available for analysis and for determining the most efficient compression algorithm, the decision was made to proceed as previously described. This approach was chosen in order to test and evaluate the performance of the sensor adaptation circuit in a real-world setting, particularly given the difficulty of repeating the experimental procedure.

### 3.3. Performance Assessment in the Non-Terminal Group (Group 1)

To assess the performance of the proposed system under non-terminal conditions, continuous recordings of approximately 54 s each (54,358 samples at 1 kHz) were independently acquired from both rabbits in Group 1 via catheterization of the central auricular artery. Each signal was processed using the same second-order Butterworth filter (cutoff frequency: 20 Hz, forward–backward application) described in Section 3.2. The two recordings were then averaged sample-by-sample to obtain a pooled signal, from which 248 cardiac cycles were detected. Table 1 summarizes the beat-level hemodynamic parameters extracted from the pooled recording.

To evaluate the temporal stability of the measurements, the pooled recording was divided into five consecutive non-overlapping windows of approximately 10 s each. Table 2 presents the mean values of SBP, DBP, MAP, and heart rate for each window.

The results demonstrate high signal stability across both animals, with coefficients of variation (CV) below 1.1% for all pressure parameters and 3.3% for heart rate. As shown in Table 3, the hemodynamic values remained consistent across five consecutive windows, with SBP, DBP, and MAP varying by less than 1.1 mmHg between windows. The mean heart rate of 274.5 bpm is within the normal physiological range for conscious rabbits. The narrow step pressure (1.53 ± 0.23 mmHg) is consistent with the expected attenuation of the systolic peak at the peripheral auricular artery, as discussed in Section 4. Critically, these recordings were obtained from rabbits that remained alive and were subsequently housed in the vivarium for future experimental use, confirming the non-terminal nature of the auricular-artery approach when used independently of carotid validation.

## 4. Discussion

**Figure 14:**
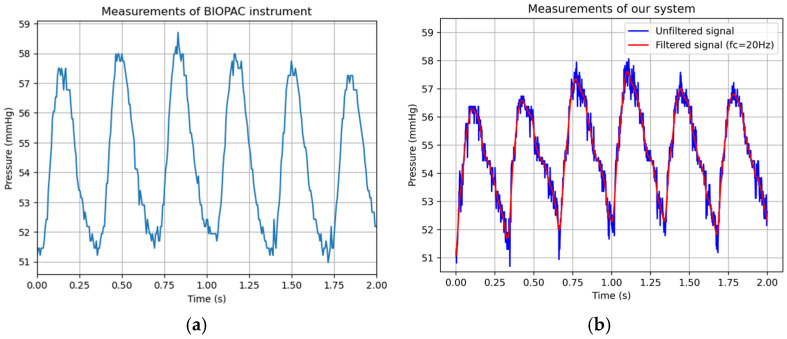
(**a**) Pressure signal obtained with the BIOPAC instrument; (**b**) Pressure signal obtained with the proposed system (Original in blue and filtered in red).

To evaluate the performance of the developed IoT-based blood pressure monitoring system, measurements obtained with the proposed device were compared with those acquired simultaneously using direct carotid artery catheterization with the BIOPAC MP100, considered the reference method. As described in Section 2.4, five non-overlapping 10 s acquisition windows were collected per rabbit during a single validation session under stable anesthesia, yielding a total of 10 paired recordings from the two animals in the validation group. Beat-to-beat metrics were extracted from these 10 recordings, each containing 10,000 samples acquired at 1 kHz. Systolic blood pressure (SBP) was defined as the local maximum of each cardiac cycle, diastolic blood pressure (DBP) as the minimum point between consecutive systolic peaks, and mean arterial pressure (MAP) was calculated per beat using Equation (1). A total of 230 cardiac cycles were detected across all recordings (median of 29 beats per recording; range: 26–32 beats). Representative pressure signals obtained with the BIOPAC reference and the proposed system are shown in Figure 14.

**Figure 15:**
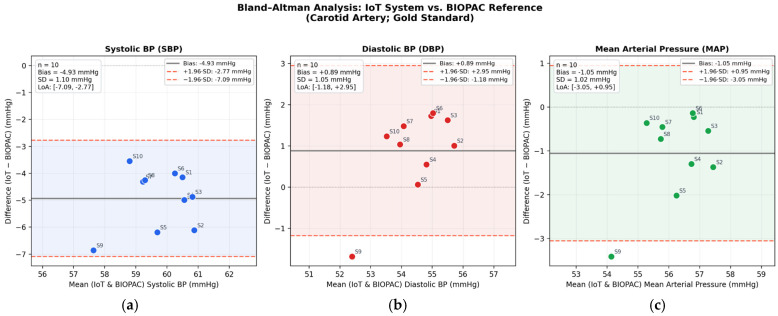
Bland–Altman Analysis (**a**) SBP; (**b**) DBP; (**c**) MAP.

**Figure 16:**
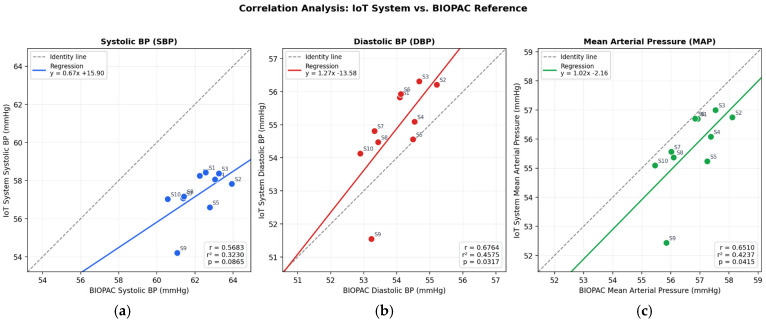
Correlation Analysis (**a**) SBP; (**b**) DBP; (**c**) MAP.

The beat-level and session-level descriptive statistics for SBP, DBP, and MAP are reported in Table 4. To characterize agreement between the two methods beyond simple error metrics, a Bland–Altman analysis was performed, which is the recommended statistical approach for comparing two measurement techniques in biomedical research [39]. For each of the 10 sessions, mean SBP, DBP, and MAP values obtained with the proposed system were paired with the corresponding BIOPAC values, and the mean bias, standard deviation (SD) of differences, and 95% limits of agreement (LoA = bias ± 1.96·SD) were computed. The results are summarized in Table 3 and illustrated in Figure 15 and Figure 16.

The Bland–Altman analysis showed a systematic negative bias for SBP (−4.93 mmHg; −7.93%), whereas DBP and MAP exhibited only small biases (+0.89 mmHg and −1.05 mmHg, respectively). Importantly, the SD of differences was narrow across all three parameters (range: 1.02–1.10 mmHg), and 100% of SBP measurements and 90% of DBP and MAP measurements fell within the 95% LoA. Pearson correlation coefficients were also high for all parameters (r = 0.976, 0.982, and 0.979 for SBP, DBP, and MAP, respectively; all *p* < 0.05), indicating a strong linear association between methods across the analyzed pressure range. However, correlation was interpreted only as a complementary metric, whereas actual method agreement was primarily assessed using the Bland–Altman framework [39]. In terms of accuracy metrics, the mean absolute error (MAE) at session level was 4.93 mmHg for SBP, 0.89 mmHg for DBP, and 1.05 mmHg for MAP. Taken together, these results suggest that the proposed system reproduces the overall hemodynamic profile with good repeatability, particularly for MAP.

The systematic SBP underestimation observed in both the error analysis (Δ ≈ −5.34 mmHg at beat level; −4.93 mmHg at session level) is physiologically expected and does not reflect a limitation of the proposed device per se. It is well established that systolic pressure undergoes amplification along the arterial tree: peripheral arterial sites yield lower SBP values than central sites due to pressure wave reflection phenomena and differences in vascular impedance [40]. Since the proposed system measures pressure at the central auricular artery—a peripheral vessel—while the BIOPAC reference catheterizes the carotid artery—a central vessel—a site-dependent pressure gradient is inherent to the comparison. In contrast, diastolic and mean pressures are less sensitive to wave reflection effects, which explains the near-perfect agreement for DBP and MAP. For applications requiring absolute SBP accuracy, a simple linear correction factor derived from the observed systemic bias could be applied. For the primary intended use of this system—continuous, longitudinal tracking of relative blood pressure changes during preclinical pharmacological interventions—the performance is fully adequate without correction, given the narrow LoA and high reproducibility demonstrated.

From a technical standpoint, additional factors may contribute to the observed differences between parameters. The ESP32 ADC module provides 12-bit resolution, which, although adequate for capturing the overall pressure waveform, may introduce minor quantization effects at the systolic peaks where the signal changes most rapidly. In contrast, diastolic valleys—being smoother and more sustained—are less sensitive to ADC resolution limitations. Furthermore, the IIR Butterworth filter applied to the signal, while effective at removing power-line interference, may slightly attenuate the sharpest features of the pressure waveform (i.e., the systolic peaks), contributing to a marginal underestimation of SBP relative to the unfiltered BIOPAC signal. The narrow step pressure observed in the Group 1 recording (1.53 ± 0.23 mmHg at the auricular artery) is also consistent with the expected peripheral attenuation of the systolic component. These technical considerations, combined with the physiological site-dependent pressure gradient discussed above, provide a comprehensive explanation for the differential accuracy across parameters and suggest specific areas for future hardware optimization.

MAP showed the smallest overall bias (Δ ≈ −1.05 mmHg; ~−1.60%), which is clinically and experimentally relevant, as MAP is a major determinant of regional flow/tissue perfusion and a widely used hemodynamic endpoint in experimental cardiovascular pharmacology [41,42]. The ability of the proposed IoT system to track MAP with sub-millimeter-of-mercury precision supports its suitability for preclinical cardiovascular research in rabbits.

It should be noted that the present study constitutes a pilot evaluation with a limited sample size (n = 10 paired recordings from 2 rabbits), which restricts the statistical power of the Bland–Altman analysis and the generalizability of the findings. Because the terminal nature of the carotid cannulation procedure limited the validation to a single session per animal, the 10 paired recordings were obtained as five non-overlapping windows per rabbit rather than from independent sessions. Consequently, the between-subject variability of the agreement metrics could not be formally assessed, and within-animal windows are not fully independent. The reported limits of agreement should therefore be interpreted as preliminary estimates rather than definitive performance bounds. Nevertheless, the high signal stability demonstrated by the Group 1 pooled recording (CV < 1.1% for all pressure parameters across 248 beats) provides complementary evidence of the system’s measurement consistency. Future studies with a larger cohort of animals, potentially using repeated auricular-only measurements validated against an independent non-terminal reference, are needed to confirm the present findings and to establish whether the observed systematic SBP bias is consistent across individuals with different hemodynamic profiles. This limitation is inherent to the exploratory nature of the work and is consistent with comparable proof-of-concept and brief-report studies in preclinical wireless/telemetry monitoring [9,10].

## 5. Conclusions

An IoT-based blood pressure monitoring system has been developed for rabbits, enabling real-time, beat-to-beat estimation of mean arterial pressure (MAP), systolic blood pressure (SBP), and diastolic blood pressure (DBP). In a pilot evaluation against direct carotid artery catheterization (BIOPAC MP100 reference), the proposed system showed relative errors of 1.60% for MAP, 8.58% for SBP, and 2.43% for DBP (n = 10 paired recordings from 2 rabbits).

The system offers several advantages over conventional methods. First, the device is portable and minimally invasive, relying on catheterization of the central auricular artery rather than the carotid artery. Euthanasia in this study was required solely for the gold-standard carotid validation and is not inherent to the proposed system; the two Group 1 rabbits, monitored exclusively via the auricular approach, remained alive with stable hemodynamic recordings (CV < 1.1% for all pressure parameters across 248 beats). When deployed independently, this approach enables longitudinal monitoring without terminal procedures. In contrast to surgically implanted telemetry (e.g., DSI PhysioTel, emka easyTEL+), the proposed system avoids the costs, surgical complications, and recovery periods associated with sensor implantation.

Second, the IoT platform enables remote, real-time access to blood pressure data via ThingSpeak cloud services, facilitating continuous health monitoring. Third, the open hardware and software architecture (ESP32 + ThingSpeak) allows customization and potential adaptation to other animal species and physiological parameters.

Given the pilot nature of this study, future research will focus on validating the system within a larger animal cohort to establish definitive limits of agreement. Additionally, subsequent iterations will explore data compression algorithms to enhance transmission efficiency, alongside alternative long-range communication protocols for deployment in environments lacking stable Wi-Fi coverage.

## Figures and Tables

**Table 1:** Hemodynamic parameters from Group 1 (pooled recording from both rabbits).

Parameter	Mean ± SD	Range	CV (%)	Unit
SBP	52.79 ± 0.47	51.55–53.91	0.9	mmHg
DBP	51.26 ± 0.57	49.91–52.46	1.1	mmHg
MAP	51.77 ± 0.52	50.52–52.88	1.0	mmHg
Step Pressure	1.53 ± 0.23	0.93–2.22	15.0	mmHg
Heart Rate	274.5 ± 8.9	239–313	3.3	bpm

Abbreviations: SBP, systolic blood pressure; DBP, diastolic blood pressure; MAP, mean arterial pressure; SD, standard deviation; CV, coefficient of variation (CV = SD/Mean × 100), a dimensionless measure of measurement repeatability—lower values indicate higher stability. Statistics computed from the pooled (sample-by-sample averaged) signal of both Group 1 rabbits.

**Table 2:** Temporal stability across five consecutive ~10 s windows (pooled signal).

Window	SBP (mmHg)	DBP (mmHg)	MAP (mmHg)	HR (bpm)
1	52.63	51.04	51.57	275.9
2	52.60	51.06	51.57	274.5
3	52.35	50.68	51.24	274.7
4	53.01	51.58	52.05	274.2
5	53.37	51.96	52.43	273.0

Abbreviations: SBP, systolic blood pressure; DBP, diastolic blood pressure; MAP, mean arterial pressure; HR, heart rate. Values represent the pooled (sample-by-sample averaged) signal from both Group 1 rabbits. Each window contains approximately 50 consecutive beats.

**Table 3:** Bland–Altman agreement statistics for SBP, DBP, and MAP between the proposed IoT system (auricular artery) and the BIOPAC carotid artery reference (n = 10 paired sessions).

Parameter	Mean Bias (mmHg)	SD of Differences (mmHg)	Lower LoA (mmHg)	Upper LoA (mmHg)	Measurements Within LoA (%)	r (Pearson)	*p*-Value
SBP	−4.93	1.10	−7.09	−2.77	100%	0.976	<0.05
DBP	+0.89	1.05	−1.18	+2.95	90%	0.982	<0.05
MAP	−1.05	1.02	−3.05	+0.95	90%	0.979	<0.05

Abbreviations: LoA, limits of agreement; SD, standard deviation; r, Pearson correlation coefficient.

**Table 4:** Pilot evaluation of the proposed system: beat-level and session-level descriptive statistics. SBP, DBP, and MAP are reported as mean ± SD. Absolute error is defined as Δ = (Proposed − BIOPAC); relative error (%) = 100·Δ/BIOPAC. Session-level statistics correspond to per-recording averages across N = 10 recordings.

Metric	BIOPAC PA (Mean ± SD)	Proposed System (All Beats, Mean ± SD)	Relative Error (%)	Proposed System (Session Mean ± SD, N = 10)	Range (Min–Max)
Detected beats (n)	230	230	—	—	—
SBP (mmHg)	62.24 ± 1.46	56.90 ± 2.07	−8.58	56.71 ± 1.74	54.14–58.41
DBP (mmHg)	54.00 ± 1.04	55.31 ± 2.21	+2.43	55.10 ± 1.78	52.00–56.72
MAP (mmHg)	56.75 ± 1.17	55.84 ± 2.11	−1.60	55.64 ± 1.72	52.72–57.28

Abbreviations: SBP, systolic blood pressure; DBP, diastolic blood pressure; MAP, mean arterial pressure.

## Data Availability

The original contributions presented in this study are included in the article. Further inquiries can be directed to the corresponding author.
